# Cumulative effects of suspended sediments, organic nutrients and temperature stress on early life history stages of the coral *Acropora tenuis*

**DOI:** 10.1038/srep44101

**Published:** 2017-03-10

**Authors:** Adriana Humanes, Gerard F. Ricardo, Bette L. Willis, Katharina E. Fabricius, Andrew P. Negri

**Affiliations:** 1 ARC Centre of Excellence for Coral Reef Studies, and College of Science and Engineering, James Cook University, 4811, Townsville, Queensland, Australia; 2 AIMS@JCU, James Cook University, Townsville, Queensland 4811, Australia; 3 Australian Institute of Marine Science, 4810, Townsville, Queensland, Australia; 4 Centre for Microscopy, Characterisation and Analysis, and UWA Oceans Institute, The University of Western Australia, Perth, Western Australia, 6009, Australia

## Abstract

Coral reproduction is vulnerable to both declining water quality and warming temperatures, with simultaneous exposures likely compounding the negative impact of each stressor. We investigated how early life processes of the coral *Acropora tenuis* respond to increasing levels of suspended sediments in combination with temperature or organic nutrients. Fertilization success and embryo development were more sensitive to suspended sediments than to high temperatures or nutrient enrichment, while larval development (after acquisition of cilia) and settlement success were predominantly affected by thermal stress. Fertilization success was reduced 80% by suspended sediments, and up to 24% by temperature, while the addition of nutrients to suspended sediments had no further impact. Larval survivorship was unaffected by any of these treatments. However, settlement success of larvae developing from treatment-exposed embryos was negatively affected by all three stressors (e.g. up to 55% by suspended sediments), while exposure only during later larval stages predominantly responded to temperature stress. Environmentally relevant levels of suspended sediments and temperature had the greatest impacts, affecting more processes than the combined impacts of sediments and nutrients. These results suggest that management strategies to maintain suspended sediments at low concentrations during coral spawning events will benefit coral recruitment, especially with warming climate.

Increasing levels of turbidity and sedimentation are contributing to coral reef degradation worldwide^1^,^2^ and are a major concern for environmental managers^3^. The introduction, resuspension and deposition of sediments in coastal marine ecosystems can be caused by natural factors (*e.g.,* waves and currents, river discharges) or human activities (*e.g.,* dredging, or enhanced terrestrial runoff of sediments due to coastal development, deforestation or poor agricultural practices). Once reaching the ocean, the fate of newly imported sediments depends on their grain size and geochemical properties^4^. Large particles are frequently deposited near the river mouth, whereas fine particles (*i.e.*, silts and clays) either flocculate or are deposited, but can also easily be resuspended, traveling 10’s of km from the source to reach inshore and mid-shelf coral reefs^5^,^6^.

Since European settlement in 1850, the development of Australia’s North Queensland catchments adjacent to the Great Barrier Reef (GBR) has led to significant changes in the quantity and quality of water discharging into the GBR lagoon^7^,^8^. Expansion of agriculture, clearing of vegetation and grazing have led to widespread soil erosion which together with increasing fertilizer application have increased river discharges of sediments, dissolved and particulate organic and inorganic nutrients, and trace elements^5^,^7^,^9^. Modelled estimates indicate that the mean annual loads of sediments delivered to the GBR lagoon have increased 5.5-fold since 1850 to 14,000 ktonnes yr^−1^ 7. Correspondingly, mean annual total nitrogen loads have increased 5.7 times to 66,000 tonnes yr^−1^, and mean annual phosphorus loads have increased 8.9 times to 14,000 tonnes yr^−1^ 7.

River discharges from catchments with significant cropping area are characterised by high concentrations of dissolved and particulate inorganic nutrients, especially nitrogen and phosphorus^7^. Inorganic nutrients from anthropogenic sources generally only persist in the GBR lagoon for periods of days to weeks^9^, as they are rapidly taken up by microbial and phytoplankton communities. They are then transformed into organic matter and undergo complex cycling between particulate and dissolved organic and inorganic forms and undergo repeated deposition-resuspension events^9^,^10^. Particulate organic nutrients reduce water clarity, stimulate microbial communities that exude mucopolysaccharides^11^ and form aggregates with sediments, thereby contributing to biological oxygen demand^12^, causing oxidative stress at the sediment/seawater interface. Organically-enriched sediments are known to increase the detrimental effects of turbidity and sedimentation on the physiology and survival of corals^2^. Decreased light availability for photosynthesis^13^, which reduces calcification^14^, productivity and growth rates, increases partial mortality, and disrupts the *Symbiodinium*-coral symbiosis^2^,^15^, are among the more serious effects that nutrient enriched sediments have on adult corals.

In addition to stress induced by elevated concentrations of sediments and nutrients, inshore coral reefs are exposed to increasing sea surface temperatures (SST) during the summer monsoonal periods^16^, compromising the health and replenishment of hard coral populations^17^. Since the beginning of the 20^th^ century, SST has risen by a global average of ~1 °C^18^ and is projected to increase by a further 2 to 3 °C by the end of the century under a moderate Representative Concentration Scenario of the Intergovernmental Panel on Climate Change (IPCC RCP 4.5^19^). Such increases in SST alone would endanger many coral species, which typically live close to their upper thermal tolerance limits^20^. It has been hypothesized that the effects of high temperatures on adult corals are ameliorated when co-occurring with suspended sediments due to reductions in light irradiance levels through shading and the provision of alternative food resources by suspended particles^21^,^22^.

The early life-history stages of corals are particularly susceptible to water quality and climate pressures^23^,^24^, and a clear understanding of the cumulative effects of these stressors on these life stages is critically important for management initiatives aimed at maintaining the resilience of reef communities^25^,^26^. Most scleractinian corals are broadcast-spawners, simultaneously releasing buoyant eggs and sperm into the water column for external fertilization^27^. Spawning and larval development of the majority of species on the GBR takes place in early austral summer (October to December)^28^, and can coincide with nutrient discharges typically driven by major river flood events during the summer monsoonal wet season (October to April)^29^. Heat stress, floods and wind-driven resuspension events of sediments can therefore co-occur and overlap with broadcast spawning, placing the sensitive early life history stages of hard corals (gametes, embryos, larvae and recruits) at risk. Despite the perception that early life history stages of corals are more sensitive to environmental change and pollution than adult stages^2^, few studies have empirically addressed their susceptibility to individual and multiple co-occurring pressures^30^,^31^,^32^,^33^,^34^,^35^,^36^,^37^.

To fill this knowledge gap, we conducted controlled experiments to examine the effects of suspended sediments, with and without nutrient enrichment, and at different temperatures, on gamete fertilization, larval survival and larval settlement of the broadcast spawning coral *Acropora tenuis.* Experiments were designed to mimic the impact of environmentally-relevant concentrations of suspended sediments originating from runoff events^29^, dredging^23^ or coastal activities^38^ under two different scenarios: i) elevated temperatures typical of those experienced during summer months^16^, and ii) nutrients typical of those produced by agricultural or coastal development activities^39^ that promote plankton blooms^10^,^40^. Results enabled the identification of differences in the sensitivity of processes that take place during the early life history stages of *A. tenuis* to single and combined effects of suspended sediments, nutrient enrichment and temperature, and allowed us to explore their potential implications for inshore coral reefs exposed to river floods, dredging and high temperatures.

## Results

### Monitoring suspended sediment, temperature and nutrient enrichment treatments

Suspended sediments remained at or very near the 5 targeted levels (0, 5, 10, 30 and 100 mg l^−1^), and seawater temperature at the 3 targeted levels (27, 30 and 32 °C) throughout four experiments (Supplementary Material Tables S1, S2, S3, S4 and S5). Organic nutrients [derived from inshore plankton and added to filtered seawater (FSW) to produce 3 levels of enrichment (+0, +0.3, and +0.6 mg organic carbon l^−1^ FSW], increased concentrations of multiple nutrients and trace elements in environmentally relevant stoichiometric ratios (Supplementary Material Table S6). Nutrient concentrations varied after incubations and among experiments, therefore the terms ‘low’, ‘medium’ and ‘high’ nutrient enrichment are used to indicate the addition of +0, +0.3, and +0.6 mg OC l^−1^ FSW.

### Experiment 1: Effects of cumulative stress conditions on gamete fertilization

**Figure 1:**
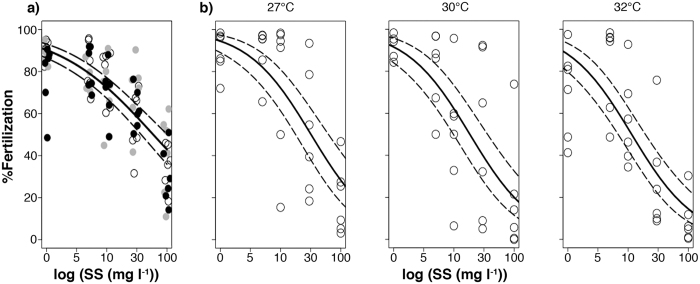
Percentage fertilization success for eggs of *Acropora tenuis* exposed to five levels of suspended sediments (0, 5, 10, 30 and 100 mg l^−1^) and either (**a**) nutrient enrichment [3 levels: low (open circles), medium (grey circles), high (black circles)] at 27 °C, or (**b**) elevated temperature (3 levels: 27, 30, and 32 °C) without organic nutrient enrichment (Experiment 1). Solid lines indicate generalised linear model fits, while dashed lines are 95% confidence intervals. Points are jittered horizontally for clarity.

At suspended sediment concentrations <10 mg l^−1^, mean fertilization success was high (70 ± 21%, mean ± s.d.) across all nutrient and temperature combinations (Fig. 1). In the absence of nutrient enrichment, the highest suspended sediment concentration (100 mg l^−1^) reduced fertilization by 59% compared to controls (p_Suspended sediments_ = <0.001, Table 1, Fig. 1a). The addition of nutrients and their interaction with suspended sediments had no significant effect on fertilization success (p_Nutrient enrichment_ = 0.113, p_Suspended sediments and nutrient enrichment_ = 0.329, Table 1, Fig. 1a). Increases in suspended sediments and temperature both had significant detrimental effects on fertilization success (p_Suspended sediments_ = <0.001, p_Temperature_ = 0.039, Table 1, Fig. 1b). No interactive effects were observed (p_Suspended sediments and temperature_ = 0.588, Table 1, Fig. 1b), indicating that when eggs were simultaneously exposed to elevated suspended sediments and temperature, impacts on fertilization were additive on a log scale. Declines in fertilization success for independent stressors in comparison to the controls ranged from ~24% in the high temperature treatment (32 °C) to 80% in the high suspended sediments treatment (100 mg l^−1^ suspended sediments). Thus suspended sediments had a greater impact on fertilization success than temperature (Supplementary Material Table S7). Suspended sediments and temperature in combination resulted in a ~89% decline in fertilization in comparison to control conditions. The concentration of suspended sediments that caused fertilization success to be reduced by 50% (IC_50_) decreased ~2-fold (from 37 to 18 mg l^−1^ suspended sediments, Supplementary Material Fig. S8, Table S9) as temperatures were increased from 27 to 32 °C.

### Experiment 2: Larval settlement after embryo and larval exposures to cumulative stress conditions

Exposure of 8-h to 36 hours old embryos during 28 hours to suspended sediments, in combination with either elevated temperatures or nutrient enrichment, during their embryological and larval development did not compromise their survivorship between the age of 36 h and settlement on day 5 (p > 0.05 for all factors and interactions, Table 1, Supplementary Material Table S7). On average, 95 ± 6% of larvae survived across all treatments in the suspended sediment and nutrient enrichment treatments, and 84 ± 15% survived in the suspended sediments and temperature treatments.

**Figure 2:**
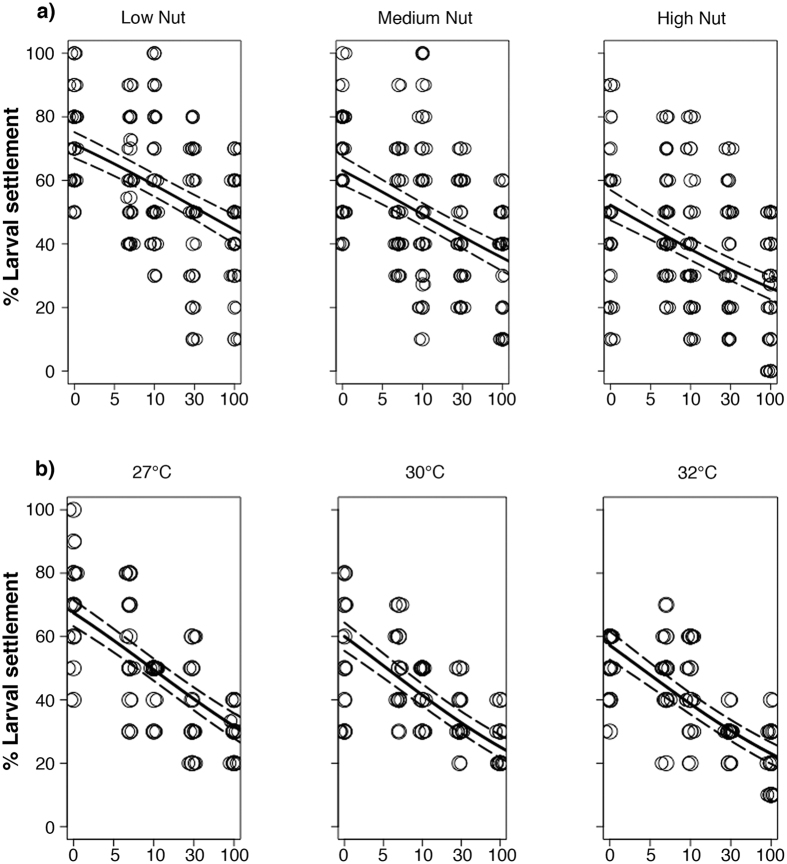
Percent larval settlement success for *Acropora tenuis* after 8-h old embryos were reared for 28 h (until ciliated) in a suspended sediment treatment (0, 5, 10, 30 or 100 mg l^−1^) combined with either (**a**) nutrient enrichment (low, medium, high) at 27 °C; or (**b**) elevated temperatures (27, 30, or 32 °C) without organic nutrient enrichment (Experiment 2). After the 28 h exposure larvae were maintained and settled under control conditions (0 mg l^−1^ suspended sediements, low nutrient enrichment and 27 °C). Solid lines indicate generalised linear model fits, while dashed lines are 95% confidence intervals. Points are jittered horizontally for clarity.

Settlement was significantly reduced for embryos exposed to high suspended sediments, temperature or nutrient enrichment (p < 0.001 for all individual factors Table 1, Fig. 2, Supplementary Material Table S7). None of the interactions were significant (p > 0.05 for both interactions, Table 1, Fig. 2, Supplementary Material Table S7), indicating that the responses of larvae exposed as embryos simultaneously to these cumulative stressors were additive on a log scale. Suspended sediments reduced settlement by up to 43 and 55% (at 100 mg l^−1^ of suspended sediments at either high nutrient enrichment or 32 °C respectively), nutrient enrichment reduced settlement by up to 33% (Fig. 2a), while temperature reduced settlement by up to 14% (Fig. 2b). Under both combinations of factors, suspended sediments had the greatest effect on larval settlement (Supplementary Material Table S7). The concentration of suspended sediments that caused a reduction in larval settlement by 50% (IC_50_) decreased with elevated nutrients and temperatures (Supplementary Material Fig. S8, Table S9). A ~5-fold decrease in IC_50_ (from suspended sediments >100 to 18 mg l^−1^) was obtained when nutrient levels increased from low to high enrichment levels (Supplementary Material Fig. S8, Table S9), while temperature caused a ~1.5-fold decrease in IC_50_ (from suspended sediments 29 to 20 mg l^−1^) with increasing temperature (from 27 to 32 °C, Supplementary Material Fig. S8, Table S9).

### Experiment 3: Larval survivorship and settlement following exposure of 3-d old larvae to cumulative stress conditions

On average, high proportions of 3-d old ciliated larvae survived the 48 h treatment exposures (97 ± 4% in the highest suspended sediment and nutrient enrichment treatments; 84 ± 22% in the highest suspended sediment and temperature treatments). Overall, there were no significant effects of suspended sediments, nutrient enrichment or temperature on larval survivorship (p > 0.05 for each individual factors and interaction, Table 1, Supplementary Material Table S7).

**Figure 3:**
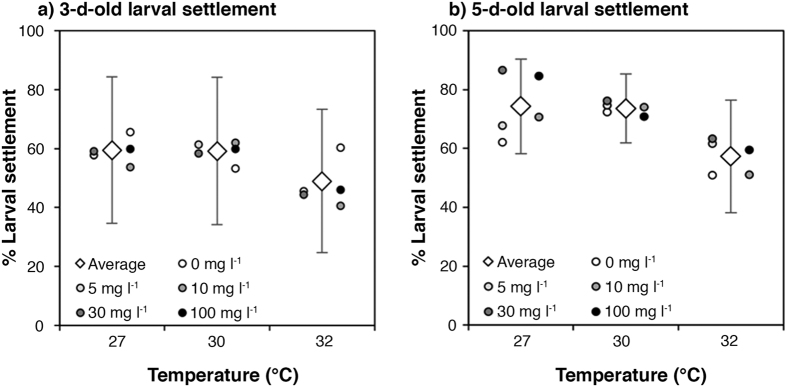
(**a**) Settlement of 3-d old larvae previously exposed for 48 h to combined suspended sediments (0, 5, 10, 30 or 100 mg l^−1^) and contrasting temperature (27, 30, or 32 °C) treatments (Experiment 3). After the exposure, larvae were settled under control conditions (0 mg l^−1^ suspended sediements, low nutrient enrichment and 27 °C). (**b**) Settlement of 5-d old larvae exposed only during settlement to suspended sediments (0, 5, 10, 30 or 100 mg l^−1^) and elevated temperatures (27, 30, or 32 °C) (Experiment 4). Diamonds indicate means at each temperature level (error bars are standard deviation), and circles indicate means for each level of suspended sediments at the corresponding temperature.

In contrast, subsequent larval settlement success was affected by high temperatures (p_Temperature_ = 0.003, Table 1, Fig. 3a, Supplementary Material Table S7), reducing it by 18% at 32 °C in comparison to control conditions. Neither suspended sediments nor nutrient enrichment, nor the combination of these two pressures had a significant effect on larval settlement (p > 0.05 for both factors and their interaction, Table 1, Supplementary Material Table S7).

### Experiment 4: Larval settlement of 5-d old larvae under cumulative stress conditions

Mean settlement success of 5-d old larvae decreased significantly when exposed to elevated temperature (p_Temperature_= 0.001, Table 1, Fig. 3b, Supplementary Material Table S7), whereas settlement success was not affected by suspended sediments or nutrient enrichment, nor by interactions between any of these three factors (p > 0.05 for both factors and all interactions, Table 1, Supplementary Material Table S7). The maximum settlement success (74 ± 16%) was obtained at 27 °C, while the minimum success (57 ± 19%) was observed at 32 °C, decreasing by 23% at the highest temperature in comparison to control conditions.

### Comparison of the effects of suspended sediments, temperature and nutrient enrichment among processes occurring in early life stages of *A. tenuis*

**Figure 4:**
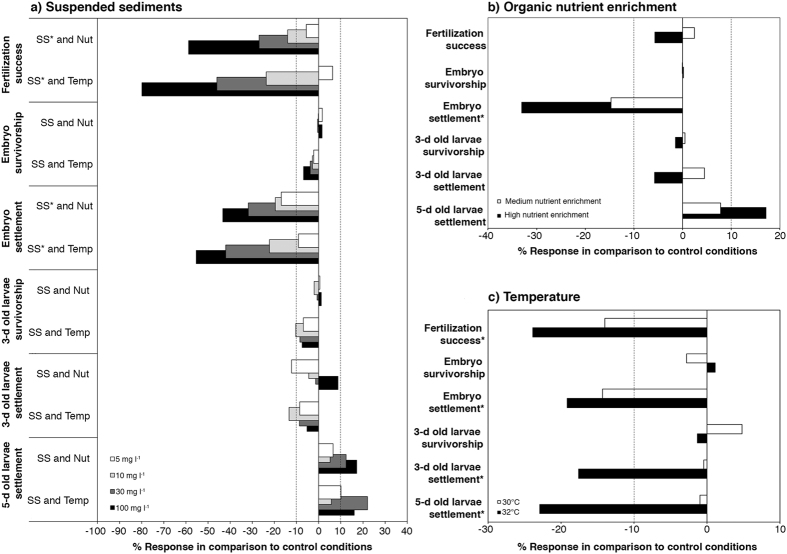
Comparative impacts of these three stressors on processes occurring during the early life history processes of *Acropora tenuis* exposed to: (**a**) suspended sediments (0, 5, 10, 30 or 100 mg l^−1^), and either high nutrients or high temperatures, (**b**) nutrient enrichment only (low, medium, high), and (**c**) a temperature treatment only (27, 30, or 32 °C). Bars represent the percent change in the response of each treatment level (main effects) compared to control conditions (0 mg l^−1^ suspended sediments, low nutrient enrichment and 27 °C). Asterisks indicate factors that were statistically significant (p < 0.05).

The effects of suspended sediments and temperature on key processes in early life history stages of *A. tenuis* were additive (i.e., interactions were not significant), when both factors had a significant effect (Table 1). Similarly, the effects of suspended sediments and nutrient enrichments were additive when both factors were significant (Table 1). Fertilization success and larval settlement when embryos were exposed to treatments during part of their development were the most affected processes, with suspended sediments causing the greatest impacts (Fig. 4a). Nutrient enrichment only affected settlement when larvae had previously been exposed to stress levels during part of their embryo development (Fig. 4b), and effect sizes were modest compared with those caused by suspended sediments. Temperature affected more processes (Fig. 4c: fertilization, settlement when embryos and larvae were exposed to elevated temperatures) than the other pressures, but the sizes of these effects were less than those caused by suspended sediments (Fig. 4a). Overall, the combination of suspended sediments aggravated by high temperatures posed the greatest risk to population replenishment of *A. tenuis,* since more processes were affected by these two stressors, and their effects were additive.

## Discussion

This study illustrates that developmental processes occurring during the early life history stages of *Acropora tenuis* differ considerably in their sensitivity to increasing levels of suspended sediments, nutrient enrichment and temperature. Fertilization and embryo development were more sensitive to suspended sediments than to high temperatures and nutrient enrichment, while larval development (following cilia acquisition) and settlement were only affected by thermal stress. No significant interactions were detected when early life history stages were exposed to two stressors simultaneously, for any of the processes evaluated, indicating additivity of responses when more than one factor had a significant effect. The effects of suspended sediments, nutrient enrichment and temperature were most pronounced on processes occurring during (i) the earliest life history stages (fertilization and embryo development), when cell division, specialization and embryo development takes place^41^, and (ii) larval settlement and the metamorphosis of motile larvae to sessile polyps, which involves extensive tissue remodeling^42^. These results suggest that environmentally realistic elevated levels of suspended sediments or high temperatures during the nights of spawning can have considerable negative impacts on the reproductive success of *A. tenuis*. Unfavorable conditions due to local (*i.e.,* wind, waves, currents, flooding or dredging) or global (*i.e.*, ocean warming) factors that increase suspended sediments and temperatures during the narrow time window when gamete fertilization, embryo development and larval settlement occur could result in reduced reproductive success of *A. tenuis* populations for that year.

Studies examining the effects of suspended sediments on coral gamete fertilization have reported decreases in fertilization success for several species^30^,^43^,^44^,^45^. The primary mechanism has recently been described for *A. tenuis,* whereby sperm coagulates with suspended particles, lowering sperm concentrations in the vicinity of eggs, hence reducing the number of sperm-egg encounters and subsequently reducing fertilization success^45^. The silt-clay sediments used in the present study are extremely cohesive and colloidal due to their physical and chemical characteristics (*e.g.* small grain sizes), readily forming flocs^6^ that are likely to attract and capture coral sperm, explaining their observed impact on fertilization success.

The effects of high temperatures on fertilization success reported here are consistent with previous studies that documented reduced fertilization success under elevated temperatures^46^,^47^. The mechanisms involved may include a reduced sperm flagella motility, which would reduce the number of sperm-egg interactions^46^, or impairment of biochemical processes involved in early cell division^48^. Nutrient enrichment had no significant effect on fertilization success in this study. Detrimental effects of nutrient enrichment on fertilization success of *A. tenuis* after exposure to combined nutrient enrichment and temperature stress have previously been reported^34^. However, the only nutrient treatments that were common between studies (suspended sediments: 0 mg l^−1^, temperature: 27 °C, nutrient enrichment: low, medium and high) had no significant effects on fertilization success in either study (Supplementary Material Table S10).

Larval survivorship was the only process that was not affected by the exposure to the treatments, even when exposures occurred before they developed cilia (8 to 36-h old). However, larval settlement success was significantly reduced when embryos were exposed to suspended sediments, revealing the latent effects that treatments can have on later developmental processes. A possible mechanism by which suspended sediments act on coral embryo development has recently been described^35^, whereby sediments cause the production of mucus by early embryos (~6-24-h old) of *A. tenuis* and *A. millepora*; however, that study did not detect subsequent effects on larval survivorship or settlement^35^. A difference between the two studies was that carbonate sediments were used in ref. 35, with an order of magnitude lower total organic carbon content than the silt-clay based sediments used in this study. Furthermore, studies are only comparable if similar embryo ages, sediment types and exposure times are investigated. Our results suggest that stressors that affect cellular function, increase energy demands, or cause mechanical abrasion during embryo development may be negative for larval settlement.

Past studies of larval settlement under conditions of sediment deposition indicate that sedimentation can mask settlement cues, obstruct settlement substrata and smother recruits (reviewed in ref. 23). Therefore, most recruits are found on vertical and downward facing surfaces or on the walls of cracks in the substrata in sediment-rich environments^49^,^50^. Since field studies record coral recruitment over time frames varying from several weeks up to 12 months after spawning^51^, it has remained unresolved whether suspended sediments reduce settlement or decrease post-settlement survivorship^2^,^52^. Our results show that under experimental conditions, high concentrations of suspended sediments (i.e., 100 mg l^−1^) do not affect larval survival and settlement success if sediments are not allowed to accumulate on the available substrata. However, although there may be no direct effect of high turbidity on settlement, sediment deposition is likely to reduce the availability of suitable substrata and lower overall settlement and recruitment success^53^,^54^.

Suspended sediments together with temperature were the factors that affected the greatest number of reproductive processes for hard corals and had the greatest proportional impacts on most early life stages. In addition, the co-occurrence of temperature with suspended sediments reduced thresholds for suspended sediments impacts (IC_50_). Similarly, elevated sediments are likely to affect thermal thresholds for early coral stages (unless these impacts are ameliorated by shading, which is uncertain for aposymbiotic gametes and larvae). These experiments were conducted under highly controlled conditions in the National Sea Simulator, allowing us to confidently assign responses to stress treatment. However, directly translating these results to field situations should be done with caution, as conditions in the field are far less consistent and may be complicated by additional factors that can affect the reproductive and recruitment process (i.e., waves, pH, salinity^30^,^55^,^56^,^57^).

Since ocean warming affects marine ecosystems at a global scale, management strategies should be directed towards minimizing dredging activities in coastal areas during periods of coral spawning, and improving water quality (i.e., suspended sediments) associated with river discharges, in order to maintain population replenishment success in inshore reefs. Assessing the risks associated with the simultaneous effects of suspended sediments and high temperatures on inshore reefs of the GBR requires further studies to determine the impacts of other sediment types in combination with temperature stress on thresholds for sensitive early life stages of corals and other tropical species. These climate-adjusted thresholds could then be incorporated into improved spatial models in order to generate effective risk maps for identification of vulnerable habitats and opportunities for management intervention.

Our results confirm that high levels of suspended sediments during periods of coral spawning affect the final reproductive success of *A. tenuis*, and indeed, are likely to affect the reproductive success of the majority of reef-building corals, which are broadcasters that release gametes synchronously during a few nights each year. We found that a high concentration of suspended sediments in the presence of high temperatures was the most detrimental combination of stressors. The effects of suspended sediments and high temperatures are likely to further increase in magnitude and change from additive to synergistic if co-occurring with additional stressors^56^ (e.g., organic nutrient enrichment^58^). Complex interactions among cumulative stressors are becoming more common on inshore reefs affected by local and global stressors^3^, and pose a significant risk for maintaining successful reproductive output required for ongoing replenishment of hard coral populations.

## Materials and Methods

### Obtaining coral gametes

Colonies of the broadcast spawning coral *Acropora tenuis* (Dana, 1846) were collected at ~6 m depth, from Davies Reefs (18°48′53′′S, 147°38′03′′E) and Esk Island (18°45′52′′S, 146°31′05′′E), both in the central mid-shelf region of the Great Barrier Reef. Gravid colonies were collected a few days before coral spawning under the permit G12/35236. Colonies were maintained in outdoor flow-through, temperature-controlled aquaria, set at the ambient seawater temperature recorded on the day of collection (27 °C), in the National Sea Simulator (SeaSim) at the Australian Institute of Marine Science (AIMS). Following spawning by seven colonies, eggs were separated from sperm using a 100 μm mesh filter and washed five times in 0.2 μm filtered sea water (FSW), as described by Negri and Heyward^59^. Gametes separated in this way were used in Experiment 1, while the remainder of the gametes were fertilized and raised in bulk flow-through cultures^59^ for Experiments 2, 3 and 4.

### Preparation of suspended sediments, temperature and nutrient enrichment treatments

Sediments were collected at Orpheus Island (18°36′39′′S, 146°29′18′′E) at 2 m depth, wet-sieved (mean ± s.d. particle size 7.3 ± 1.5 μm, 95% <20 μm) and kept in 60 l tanks with flow-through seawater at 27 °C. Suspended sediment treatments (0, 5, 10, 30 or 100 mg l^−1^) were prepared through dilution with FSW and adjusted with a nephelometer (TPS 90FL-T). Two replicate Schott bottles (2 l) per treatment, containing the sediments, were incubated for 3 h at the treatment temperatures prior to the start of each experiment (Supplementary Material Table S5).

For the organic nutrient treatments, FSW was enriched with decaying natural inshore plankton and suspended detritus to maintain a realistic stoichiometric composition of nutrients and trace elements. Plankton was collected at reefs around Orpheus Island with a 100-μm plankton net, sieved to remove >26 μm fragments, blended and frozen until use. After determination of total organic carbon (OC) in the stock solution, three nutrient enrichment treatments were prepared with nominal concentrations of +0, +0.3, or +0.6 mg OC l^−1^ FSW. The nutrients were added to two replicate Schott bottles (2 l) per treatment, which contained the suspended sediments. Bottles were incubated for 48 h at 200 μmol photons m^−2^ s^−1^ light intensity (12 h:12 h diurnal cycle) at 27 °C to promote development of natural microbial communities, uptake of bioavailable fraction of inorganic nutrients and their transformation into organic nutrients. Decaying plankton was previously used to study nutrient effects on corals^15^,^34^, and incubations were long enough to stimulate microbial community development, uptake of dissolved inorganic nutrients and conversion into organic matter during natural suspension events^60^,^61^,^62^.

Experimental suspended sediment concentrations were within the range of conditions measured on inshore GBR reefs during wind suspension or associated with dredging projects. Concentrations up to 5 mg l^−1^ occur under calm conditions on inshore reefs^63^, concentrations between 5–30 mg l^−1^ are typical after storms and river plumes, and concentrations up to 100 mg l^−1^ have been recorded close to seafloor dredging activities^38^,^45^,^64^,^65^. Nutrient enrichment concentrations were based on measurements in the inshore GBR^7^,^39^,^66^ (Table S3).

Control treatments were 0 mg l^−1^ suspended sediments without addition of OC (+0 mg OC l^−1^ FSW) at 27 °C. To characterize the different treatments, concentrations of suspended sediments, total organic carbon, dissolved organic carbon, total dissolved phosphorus, dissolved organic nitrogen, total particulate nitrogen, ammonium, phosphate, nitrate and nitrite, were measured once in duplicate subsamples at the end of each incubation in each replicate (Schott bottles). Water samples were analyzed by the Analytical Services laboratory at AIMS following Schaffelke *et al*.^39^.

### Experiment 1: Effects of cumulative stress conditions on gamete fertilization

**Figure 5:**
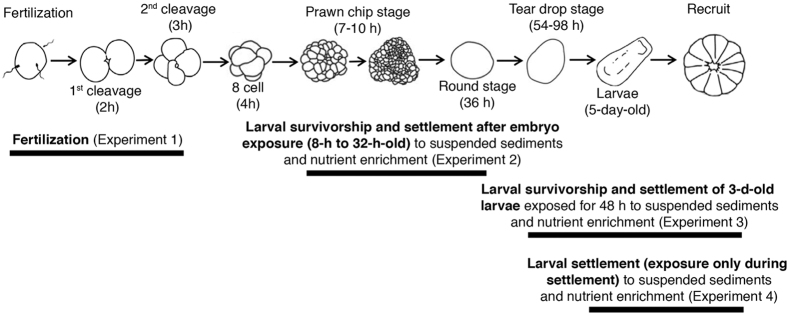
Experiments performed with early life history stages of *Acropora tenuis* exposed to suspended sediments (SS: 0, 5, 10, 30 or 100 mg l^−1^) in combination with either (**a**) a nutrient enrichment treatment (low, medium, high), or (**b**) a temperature treatment (27, 30 or 32 °C) treatments. Black bars indicate the processes or stages involved in each experiment (modified from Jones *et al*.^23^).

To test the impacts of suspended sediments, nutrients and temperature on gamete fertilization, 5 levels of suspended sediments (0, 5, 10, 30 or 100 mg l^−1^) were combined with either 3 levels of nutrients (+0, +0.3, or +0.6 mg OC l^−1^ FSW), or 3 levels of temperature (27, 30, or 32 °C) to produce 30 experimental treatments (15 for the suspended sediments and nutrient enrichment experiment; and 15 for the suspended sediments and temperature experiment; Fig. 5). For each treatment combination, twelve replicate 50 ml clear polypropylene chambers were prepared, each containing 25 ml of the modified seawater. Eggs were added to half of the chambers (~200 eggs per chamber), and sperm aliquots to the other half. Chambers were transferred into temperature incubators at the designated temperature (Supplementary Material Table S5). After 30 min, sperm and egg chambers were combined to initiate fertilization, resulting in a total of 180 chambers across the 30 treatments (6 per treatment). The final sperm concentration was 5 × 10^4^ ml^−1^, being slightly suboptimal for maximum fertilization^67^, thereby increasing the sensitivity of the assay^45^,^68^. Chambers were returned into incubators, and gently shaken every 30 minutes to maintain sediments suspended (Supplementary Material Table S1). When the second cleavage was observed (1.5 h after fertilization), 2 ml of zinc formalin fixative (Z-fix preservative, Anatech Limited) were added to terminate embryo development. Fertilization success (proportion of eggs fertilized, Supplementary Material Table S11) was assessed under a stereomicroscope.

### Experiment 2: Larval settlement after embryo and larval exposures to cumulative stress conditions

Embryos fertilized under control conditions were exposed to the 30 treatment combinations from the early gastrula stage (8-h old) until they were ciliated larvae (~36-h old; Fig. 5). Six water baths (400 l) were used to maintain seawater temperatures (Supplementary Material Table S2). For each treatment, four replicate experimental systems (Supplementary Material S12 and Fig. S13) were set up and ∼250 embryos added to each experimental tank. Suspended sediment concentrations were derived from turbidity readings taken every 6 h in each experimental tank (Supplementary Material Table S2). After 28 h larvae were transferred to six-well polystyrene tissue culture plates (NuncTM, Denmark) maintained at 27 °C in a temperature-controlled room. Each well contained 10 ml FSW at 27 °C and 10 larvae (n = 12 wells for each suspended sediment and nutrient enrichment combination, and n = 24 wells for each suspended sediment and temperature combination). When larvae where 5-d old, 2-mm^2^ chips of live crustose coralline algae (CCA) *Porolithon onkodes* were added to induce larval settlement^69^. After 24 h, the number of settled larvae in each well was recorded (Supplementary Material Table S11).

### Experiment 3: Larval survivorship and settlement following exposure of 3-d old larvae to cumulative stress conditions

For each of the 30 treatment combinations, six replicate 100 ml clear polystyrene chambers were prepared containing 80 ml of the appropriately modified seawater and 3-d old larvae (n = 20 larvae per replicate, Fig. 5). Chambers were placed in mechanical rollers to keep sediments suspended (Supplementary Material Table S3). Rollers were kept in temperature incubators to maintain target temperatures (Supplementary Material Table S5). Larvae were exposed to treatment conditions for 48 h, with one water change after 24 h. After exposure, larvae from each replicate were counted and transferred to six-well polystyrene tissue culture plates (NuncTM, Denmark, n = 6 replicate wells per treatment, n = 10 larvae per well). CCA chips were added to each well and settlement success was assessed after 24 h (Supplementary Material Table S11).

### Experiment 4: Larval settlement of 5-d old larvae under cumulative stress conditions

The experimental setup from Experiment 2 was used to expose 5-d old larvae to each of the 30 treatment combinations (Fig. 5, Supplementary Material Tables S4, S5). Larvae (n = 150) were added to each experimental tank, which also contained 15 aragonite substrata (~20 mm diameter) previously conditioned with CCA (Supplementary Material S12 and Fig. S13). The aragonite plugs were suspended in the experimental tanks, with the CCA-colonized surface facing downwards to prevent sediment accumulation. After 24 h, the number of settlers per plug was assessed under a stereomicroscope (Supplementary Material Table S11).

### Data analysis

Generalized linear models (GLM) were used to assess changes in fertilization success, larval survivorship and settlement as a function of the three fixed factors: suspended sediments (numerical factor: 0, 5, 10, 30 or 100 mg l^−1^), nutrient enrichment (categorical factor: low, medium or high organic nutrients) or temperature (categorical factor: control, low stress or moderate stress). Quasi-binomial errors and the log-link function were used when the model had overdispersion. All calculations were conducted using the package lme4 in R (R Development Core Team, 2016).

Data were further analyzed when more than one factor was significant (GLM), using nonlinear regression curves (four-parameter logistic models) using GraphPad Pism (v.7), to derive effective concentration point intercepts (i.e. EC_50_ values) for the various treatment combinations^70^. All curves were tested for normality of the residuals and a replicate test was applied to assess goodness-of-fit. Absolute EC_50_ values were determined using a four-parameter logistic model. All models were interpolated to 50% of the ‘top’ (EC_50_) of the control temperature (27 °C) or control nutrient concentration (low) treatments. In Experiment 2, 95% confidence intervals could not be determined for larval settlement success in the medium nutrient enrichment treatment because of ambiguity in the estimation of parameters.

## Additional Information

**How to cite this article:** Humanes, A. *et al*. Cumulative effects of suspended sediments, organic nutrients and temperature stress on early life history stages of the coral *Acropora tenuis. Sci. Rep.* **7**, 44101; doi: 10.1038/srep44101 (2017).

**Publisher's note:** Springer Nature remains neutral with regard to jurisdictional claims in published maps and institutional affiliations.

## Supplementary Material

Supplementary Material

## Figures and Tables

**Table 1:** Treatment effects on processes occurring during early life history stages of *Acropora tenuis*.

Life history stage	Dependent variable	Summary of findings	p-values					
			SS	Nut	SS × Nut	SS	Temp	SS × Temp
Gametes	1. Fertilization	1. SS decreased fertilization	**<0.001**	0.113	0.329			
		2. SS, Temp both decreased fertilization				**<0.001**	**0.039**	0.588
Embryos	2.a. Survivorship	1. No effect of SS or Nut	0.659	0.938	0.523			
		2. No effect of SS or Temp				0.078	0.416	0.809
	2.b. Settlement	1. SS, Nut both decreased settlement	**<0.001**	**<0.001**	0.577			
		2. SS, Temp both decreased settlement				**<0.001**	**<0.001**	0.112
Larvae	3.a. Survivorship	1. No effect of SS or Nut	0.444	0.135	0.784			
		2. No effect of SS or Temp				0.869	0.356	0.140
	3.b. Settlement	1. No effect of SS or Nut	0.613	0.448	0.487			
		2. Temp decreased settlement				0.269	**0.028**	0.271
Settlement	Settlement	1. No effect of SS or Nut	0.302	0.456	0.280			
		2. Temp decreased settlement				0.054	**0.001**	0.121

Analyses are based on generalised linear models, with five concentrations of suspended sediments (SS: 0, 5, 10, 30 or 100 mg l^−1^), three levels of nutrient enrichment (Nut: low, medium, high) and three temperatures (Temp: 27, 30, or 32 °C), each treated as a fixed factor. All experiments combined. Significant relationships (at p < 0.05) are printed in bold. Refer to Table S10 for more detailed information for each GLM analysis.
