# Supplementary material for: Cumulative effects of suspended sediments, organic nutrients and temperature stress on early life history stages of the coral Acropora tenuis
Source: Sci Rep. 2017 Mar 10;7:44101. doi: 10.1038/srep44101 (PMC5345069; doi:10.1038/srep44101)
Supplement: Supplementary Material [file srep44101-s1.pdf]

Supplementary Material

Cumulative effects of suspended solids, organic nutrients and temperature stress on early life history stages of the coral *Acropora tenuis*

Adriana, Humanes[1,2*]; Gerard F., Ricardo[2,3,4]; Bette L. Willis[1]; Katharina E. Fabricius[2]; Andrew P. Negri[2]

*Corresponding author.

email: adrihumanes@gmail.com

Table S1: Concentration of suspended sediments (0, 5, 10, 30 and 100 mg l^{-1}) used in Experiment 1 (mean ± s.d.).

Suspended sediments (mg l^{-1})	Initial	30 min	60 min	90 min	120 min	150 min
**0**	0.1±0.1	0.2±0.6	0.2±0.4	0.4±0.3	0.4±0.2	0.4±0.1
**5**	5.6±0.5	4.4±0.5	4.8±0.9	4.8±0.1	4.4±0.5	5.1±1.0
**10**	10.8±0.5	10.4±0.5	9.4±0.5	8.3±1.0	7.2±0.5	7.6±1.0
**30**	30.4±0.9	29.6±1.3	29.7±0.5	27.9±0.5	27.2±1.5	25.7±1.1
**100**	103.6±1.0	101.5±0.5	95.4±2.3	96.9±6.3	94.0±1.8	93.3±2.3

Table S2: Concentration of suspended sediments (0, 5, 10, 30 and 100 mg l^{-1}) used in Experiment 2 (mean ± s.d.). Measurements were performed before starting the experiment (initial) and every 6 h until the end of the experiment.

**Suspended sediments (mg l^{-1})**	**Initial**	**6 h**	**12 h**	**18 h**
**0**	0.1±0.2	0.2±0.3	0.8±0.6	0.4±0.5
**5**	5.5±1.0	4.0±0.8	3.4±1.0	4.3±1.0
**10**	10.6±2.3	10.5±1.3	8.7±0.8	7.2±1.0
**30**	29.5±1.7	27.8±3.4	25.7±1.3	20.7±1.7
**100**	102.3±2.3	94.4±1.7	89.6±1.4	87.4±2.6

Table S3: Concentration of suspended sediments (0, 5, 10, 30 and 100 mg l^{-1}) used in Experiment 3 (mean ± s.d.). Measurements were performed before starting the experiment (initial), before the water change (24 h) and before finishing the experiment (48 h).

**Suspended sediments (mg l^{-1})**	**Initial**	**24 h**	**48 h**
**0**	0.2±0.6	0.5±0.9	0.8±0.9
**5**	7.6±1.0	1.9±0.5	1.2±1.0
**10**	12.6±0.5	5.5±0.5	4.0±1.3
**30**	34.6±0.9	21.8±0.9	19.7±1.7
**100**	110.7±1.3	83.0±1.3	86.2±2.9

Table S4: Concentration of suspended sediments (0, 5, 10, 30 and 100 mg l^{-1}) used in Experiment 4 (mean ± s.d.). Measurements were performed before starting the experiment (initial) and every 6 hours until the end of the experiment.

Suspended sediments (mg l^{-1})	Initial	6 h	12 h	18 h
**0**	0.5±0.5	0.5±0.6	0.9±0.9	0.5±0.8
**5**	7.2±1.0	4.9±1.0	3.1±1.0	2.3±0.6
**10**	13.1±1.8	10.5±1.3	7.5±1.4	4.6±0.6
**30**	33.2±0.9	30.3±1.0	28.0±1.3	23.3±1.3
**100**	107.9±1.0	105.9±0.9	102.9±2.1	86.8±2.3

Table S5: Temperatures (°C) during the performance of the Experiments 1, 2, 3 and 4 (mean ± s.d.). SS: suspended sediments, Nut: nutrient enrichment, Temp: temperature.

		Temperature obtained			
**Factors combination**	**Target temperature**	**Fertilization**	**Embryos**	**Larvae**	**Settlement**
SS x Nut	27°C	27.36±0.21	27.12±0.24	27.34±0.12	27.22±0.29
SS x Temp	27°C	27.34±0.36	27.18±0.05	27.26±0.16	27.37±0.13
	30°C	29.98±0.57	29.61±0.13	29.82±0.20	30.03±0.07
	32°C	32.11±0.36	31.17±0.14	31.92±0.43	31.49±0.25

Table S6: Water quality parameters for the different suspended sediments concentrations (mg l^{-1}) and nutrient enrichment (low in white, high in dark grey) at the start of each experiment. Values shown are means and standard deviations. Number of replicates: 2 per water quality factor and treatment. Ranges from seawater values from the inshore of the Great Barrier Reef are added for comparison [1].

Experiment	Suspended sediments	Nutrient enrichment	DOC (µM)	TOC (µM)	NH_4 (µM)	NO_2+NO_3 (µM)	NO_2 (µM)	TDN (µM)	TN (µM)	PO_4 (µM)	TDP (µM)
Schaffelke et al 2012			42.8-195.6	3.9-70.5	0-0.82	NA	NA	2.2-11.5	0.5-2.7	0.02-0.6	0-1.01
1	0	Low	88.4±1.7	16.6±3.0	0.6±0.1	0.7±0.1	0.2±0.1	9.6±0.6	3.1±0.9	0.1±0.1	0.2±0.1
		Medium	86.8±1.6	33.2±8.1	5.5±2.2	0.8±0.1	0.2±0.1	17.9±2.9	6.7±1.2	0.1±0.1	0.3±0.1
		High	103.1±27.2	33.7±3.3	6.0±1.5	0.8±0.1	0.2±0.1	18.7±1.9	6.3±0.2	0.2±0.1	0.4±0.2
	5	Low	81.1±2.9	36.1±3.5	0.8±0.1	0.8±0.1	0.2±0.1	12.2±0.1	4.8±0.4	0.1±0.1	0.2±0.1
		Medium	87.0±1.7	54.8±5.4	4.0±0.9	0.8±0.1	0.2±0.1	19.9±1.5	10.1±0.7	0.1±0.1	0.2±0.1
		High	105.4±18.3	65.4±5.5	6.2±0.2	0.8±0.1	0.2±0.1	21.6±1.2	10.8±1.9	0.1±0.1	0.3±0.1
	10	Low	79.1±3.8	46.2±4.1	0.7±0.0	0.6±0.1	0.2±0.1	11.4±0.9	7.1±1.2	0.1±0.1	0.4±0.3
		Medium	82.2±0.5	62.9±0.1	2.5±0.2	0.8±0.1	0.2±0.1	15.2±0.3	9.4±0.9	0.1±0.1	0.2±0.1
		High	100.4±4.1	66.2±1.5	5.9±0.1	0.8±0.1	0.2±0.1	19.1±0.8	11.4±1.3	0.1±0.1	0.3±0.1
	30	Low	88.6±2.2	108.8±42.1	0.7±0.1	0.5±0.1	0.2±0.1	12.0±0.2	15.0±4.0	0.1±0.1	0.2±0.1
		Medium	90.4±0.6	132.6±12.3	2.5±0.2	0.7±0.1	0.2±0.1	14.7±1.5	22.3±3.3	0.1±0.1	0.2±0.1
		High	108.4±18.7	201.2±30.9	5.2±0.5	0.8±0.1	0.2±0.1	19.5±0.4	33.4±6.1	0.1±0.1	0.3±0.1
	100	Low	91.4±0.2	NA	0.7±0.1	0.2±0.1	0.1±0.1	11.2±0.4	NA	0.1±0.1	0.2±0.1
		Medium	96.9±15.0	317.7±55.2	1.9±0.1	0.7±0.1	0.2±0.1	13.5±0.5	43.9±1.1	0.1±0.1	0.2±0.1
		High	97.9±11.3	313.1±17.2	4.1±0.6	0.8±0.1	0.2±0.1	17.6±0.5	45.2±3.3	0.1±0.1	0.2±0.1
2	0	Low	109.2±17.4	53.3±32.8	0.2±0.2	0.4±0.3	0.2±0.1	12.7±2.2	6.7±2.2	0.3±0.2	0.1±0.1
		Medium	115.7±8.7	101.2±56.5	0.2±0.2	0.4±0.3	0.2±0.1	14.7±4.0	10.3±3.3	0.3±0.1	0.1±0.1
		High	124.8±24.9	116.9±103.7	0.2±0.2	0.4±0.3	0.2±0.1	15.9±5.0	11.1±9.2	0.5±0.3	0.4±0.5
	5	Low	103.0±15.4	49.7±10.7	0.1±0.1	0.2±0.1	0.2±0.1	12.5±2.1	7.5±0.7	0.3±0.1	0.1±0.1
		Medium	114.2±3.6	161.6±17.3	0.2±0.2	0.3±0.3	0.2±0.1	16.8±2.1	20.7±1.0	0.4±0.3	0.1±0.1
		High	158.4±41.6	279.2±119.8	0.2±0.2	1.0±0.9	0.2±0.1	21.3±2.7	27.6±13.0	0.1±0.1	0.3±0.4
	10	Low	104.2±4.8	91.8±37.6	0.1±0.1	0.2±0.1	0.2±0.1	12.5±0.8	13.2±5.4	0.5±0.4	0.1±0.1
		Medium	111.3±13.5	114.9±7.6	0.1±0.1	0.4±0.3	0.2±0.1	13.7±2.3	17.3±3.0	0.2±0.1	0.1±0.1
		High	125.7±13.1	176.7±92.1	0.1±0.1	0.5±0.3	0.5±0.3	13.1±2.2	19.8±8.5	0.2±0.1	0.3±0.1
	30	Low	100.1±2.5	279.0±82.6	0.1±0.1	0.3±0.2	0.2±0.1	16.5±4.5	32.1±13.9	0.2±0.1	0.1±0.1
		Medium	104.5±8.9	296.3±104.6	0.2±0.2	0.3±0.3	0.2±0.1	17.8±6.8	41.2±8.2	0.3±0.1	0.2±0.1
		High	165.8±121.3	392.7±163.5	0.4±0.2	0.4±0.3	0.2±0.1	20.0±4.7	48.1±22.7	0.3±0.2	0.3±0.1
	100	Low	94.9±7.3	267.4±80.5	0.1±0.1	0.7±0.3	0.2±0.1	13.3±1.6	31.6±8.4	0.2±0.1	0.2±0.1
		Medium	106.4±26.4	295.8±29.4	0.1±0.1	0.7±0.2	0.2±0.1	14.2±3.3	36.9±8.8	0.3±0.1	0.2±0.1
		High	134.4±54.5	352.5±108.7	0.1±0.1	0.8±0.2	0.2±0.1	17.7±4.3	38.7±3.2	0.3±0.2	0.2±0.1

Table S6 (continued):

Experiment	Suspended sediments	Nutrient enrichment	DOC (μM)	TOC (μM)	NH_4 (μM)	NO_2+NO_3 (μM)	NO_2 (μM)	TDN (μM)	TN (μM)	PO_4 (μM)	TDP (μM)
3	0	Low	84.8±14.0	16.9±1.5	0.9±0.1	1.3±0.1	0.3±0.1	17.2±1.4	1.6±0.1	0.1±0.1	0.5±0.1
		Medium	97.8±12.4	30.9±0.2	3.0±0.3	1.4±0.1	0.3±0.1	16.8±0.1	4.1±0.2	0.2±0.1	0.5±0.1
		High	103.9±2.4	36.2±1.5	5.6±0.1	1.4±0.1	0.3±0.1	19.2±0.7	5.9±0.4	0.4±0.1	0.7±0.1
	5	Low	104.6±10.7	39.8±12.9	0.8±0.1	1.2±0.1	0.3±0.1	13.3±2.3	4.1±0.9	0.1±0.1	0.3±0.1
		Medium	109.2±5.9	54.4±0.3	1.8±0.1	1.3±0.1	0.3±0.1	14.2±2.5	7.4±0.2	0.1±0.1	0.4±0.1
		High	129.4±5.7	83.1±6.1	4.7±0.6	1.3±0.1	0.3±0.1	20.5±0.1	14.6±0.4	0.2±0.1	0.5±0.1
	10	Low	90.5±0.6	49.0±7.4	0.8±0.1	1.2±0.1	0.3±0.1	12.2±0.1	4.9±0.5	0.1±0.1	0.3±0.1
		Medium	119.4±0.6	56.3±8.9	1.6±0.1	1.3±0.1	0.3±0.1	16.0±1.0	6.6±1.5	0.1±0.1	0.4±0.1
		High	125.7±24.0	85.3±2.7	4.2±0.2	1.4±0.1	0.3±0.1	23.2±0.6	14.0±1.2	0.1±0.1	0.5±0.1
	30	Low	123.4±6.7	120.2±2.3	0.9±0.1	1.0±0.1	0.3±0.1	13.1±0.5	14.5±5.6	0.1±0.1	0.3±0.1
		Medium	138.9±21.1	141.4±0.9	2.1±0.4	1.4±0.1	0.3±0.1	23.6±3.9	15.2±1.7	0.1±0.1	0.3±0.1
		High	241.5±133.4	173.2±115.2	6.2±0.3	1.4±0.1	0.3±0.1	25.5±0.7	19.7±2.4	0.1±0.1	0.5±0.1
	100	Low	131.8±14.6	243.6±16.2	0.8±0.1	0.7±0.2	0.3±0.1	13.1±0.1	25.9±1.4	0.1±0.1	0.3±0.1
		Medium	153.4±23.1	278.9±15.3	1.4±0.4	1.4±0.1	0.3±0.1	19.0±1.8	31.2±0.1	0.1±0.1	0.4±0.1
		High	154.8±27.3	298.2±26.8	5.7±0.3	1.5±0.1	0.3±0.1	26.4±2.4	33.8±1.5	0.2±0.1	0.5±0.1
4	0	Low	101.3±9.6	21.8±5.0	2.0±1.4	0.7±0.3	1.4±1.3	22.7±3.3	4.5±0.8	0.2±0.1	0.5±0.1
		Medium	112.0±3.8	25.7±1.3	4.6±3.4	0.7±0.2	1.7±0.1	24.7±0.6	5.3±0.2	0.2±0.1	0.6±0.1
		High	143.3±59.4	26.4±8.4	4.2±2.0	0.8±0.2	2.0±0.5	25.3±3.2	5.9±2.1	0.2±0.1	0.6±0.1
	5	Low	101.9±13.1	67.8±9.9	0.9±1.0	0.4±0.1	0.2±0.1	14.3±1.7	9.3±10.6	0.2±0.1	0.5±0.1
		Medium	109.2±4.2	77.3±3.4	1.4±1.1	0.4±0.1	0.2±0.1	18.9±0.9	10.6±2.4	0.2±0.1	0.5±0.1
		High	110.6±3.7	80.0±14.5	2.4±1.5	1.6±1.2	0.7±0.6	19.9±1.4	14.1±0.5	0.2±0.1	0.6±0.1
	10	Low	94.8±9.4	70.3±12.3	1.4±1.0	0.3±0.1	0.2±0.1	14.2±0.6	9.1±1.4	0.2±0.1	0.5±0.1
		Medium	110.5±5.8	81.4±5.7	1.3±0.9	1.7±1.5	0.7±0.5	13.9±2.3	11.8±0.8	0.2±0.1	0.6±0.1
		High	121.3±6.2	104.4±49.4	3.2±2.3	4.5±4.9	1.3±1.3	22.9±9.3	16.1±6.3	0.2±0.1	0.6±0.1
	30	Low	99.6±21.2	225.5±42.7	3.0±1.8	1.9±1.4	0.8±0.6	19.9±5.2	32.8±4.1	0.2±0.1	0.6±0.1
		Medium	110.6±18.2	343.3±34.8	3.2±1.4	3.1±2.1	1.0±0.6	19.1±2.3	48.1±9.2	0.2±0.1	0.7±0.2
		High	104.4±12.5	341.6±98.2	3.8±3.7	3.1±3.1	1.1±1.0	21.1±6.9	52.3±16.4	0.2±0.1	1.3±1.4
	100	Low	102.2±5.6	226.1±13.5	0.5±0.6	0.4±0.2	0.2±0.1	14.1±1.0	36.4±3.2	0.2±0.1	0.5±0.1
		Medium	112.5±16.7	236.1±16.9	0.9±0.8	0.3±0.3	0.2±0.1	15.3±3.9	39.3±8.1	0.2±0.1	0.6±0.1
		High	122.2±24.1	301.5±64.6	1.0±1.6	0.4±0.3	0.9±0.7	16.8±4.8	43.9±4.9	0.2±0.1	0.6±0.1

Table S7: Results of generalized linear models (GLM) of the effects of suspended sediments (SS: 0, 5, 10, 30 or 100 mg l^{-1}) and a) nutrient enrichment (Nut: low, medium, high), or b) contrasting temperatures (Temp: 27, 30, or 32°C) on early life history processes (gamete fertilization, larvae survivorship and settlement) of *Acropora tenuis*. Suspended sediments, nutrient enrichment and temperature were treated as fixed factors.

**Experiment**	**Factor**	**Df**	**Deviance**	**F-value**	**p-value**
Fertilization (Experiment 1)	SS	1	5864.2	139.92	<**0.001**
	Nut	2	2348.0	2.22	0.113
	SS x Nut	2	2232	1.12	0.329
	SS	1	9270.9	88.07	<**0.001**
	Temp	2	4912.3	3.35	**0.039**
	SS x Temp	2	4553.0	0.55	0.588
Larvae survivorship (Experiment 2)	SS	1	332.6	0.19	0.659
	Nut	2	332.6	0.06	0.938
	SS x Nut	2	332.4	0.64	0.523
	SS	1	261.1	3.14	0.077
	Temp	2	259.1	0.88	0.416
	SS x Temp	2	256.5	0.21	0.951
Larvae settlement (Experiment 2)	SS	1	696.1	72.75	<**0.001**
	Nut	2	666.6	27.36	<**0.001**
	SS x Nut	2	576.9	0.55	0.577
	SS	1	202.5	143.87	<**0.001**
	Temp	2	123.7	9.69	<**0.001**
	SS x Temp	2	111.4	2.21	0.112

Table S7 (continued):

Experiment	Factor	Df	Deviance	F-value	p-value
Larvae survivorship (Experiment 3)	SS	1	119.1	0.58	0.444
	Nut	2	123.2	2.03	0.135
	SS x Nut	2	118.4	0.24	0.784
	SS	1	428.0	0.02	0.869
	Temp	2	432.6	1.03	0.356
	SS x Temp	2	428.0	1.98	0.140
Larvae settlement (Experiment 3)	SS	1	335.0	0.25	0.613
	Nut	2	339.5	0.80	0.448
	SS x Nut	2	334.2	0.72	0.487
	SS	1	553.7	1.22	0.269
	Temp	2	570.7	3.60	**0.028**
	SS x Temp	2	550.2	1.31	0.271
Larvae settlement (Experiment 4)	SS	1	6.8	1.08	0.302
	Nut	2	6.9	0.79	0.456
	SS x Nut	2	6.7	1.30	0.280
	SS	1	6.8	3.86	0.054
	Temp	2	8.0	7.42	**0.001**
	SS x Temp	2	6.3	2.18	0.121

Figure S8: *Acropora tenuis* inhibition of a) gamete fertilization and b-c) settlement of embryos exposed to treatment conditions during embryo development. Curves fitted to four-parameter logistic models plotted over a range of suspended sediments (SS: 0, 5, 10, 30 or 100 mg l^{-1}) and a-b) contrasting temperatures (Temp: 27, 30, or 32°C) or c) nutrient enrichment (Nut: low, medium, high).

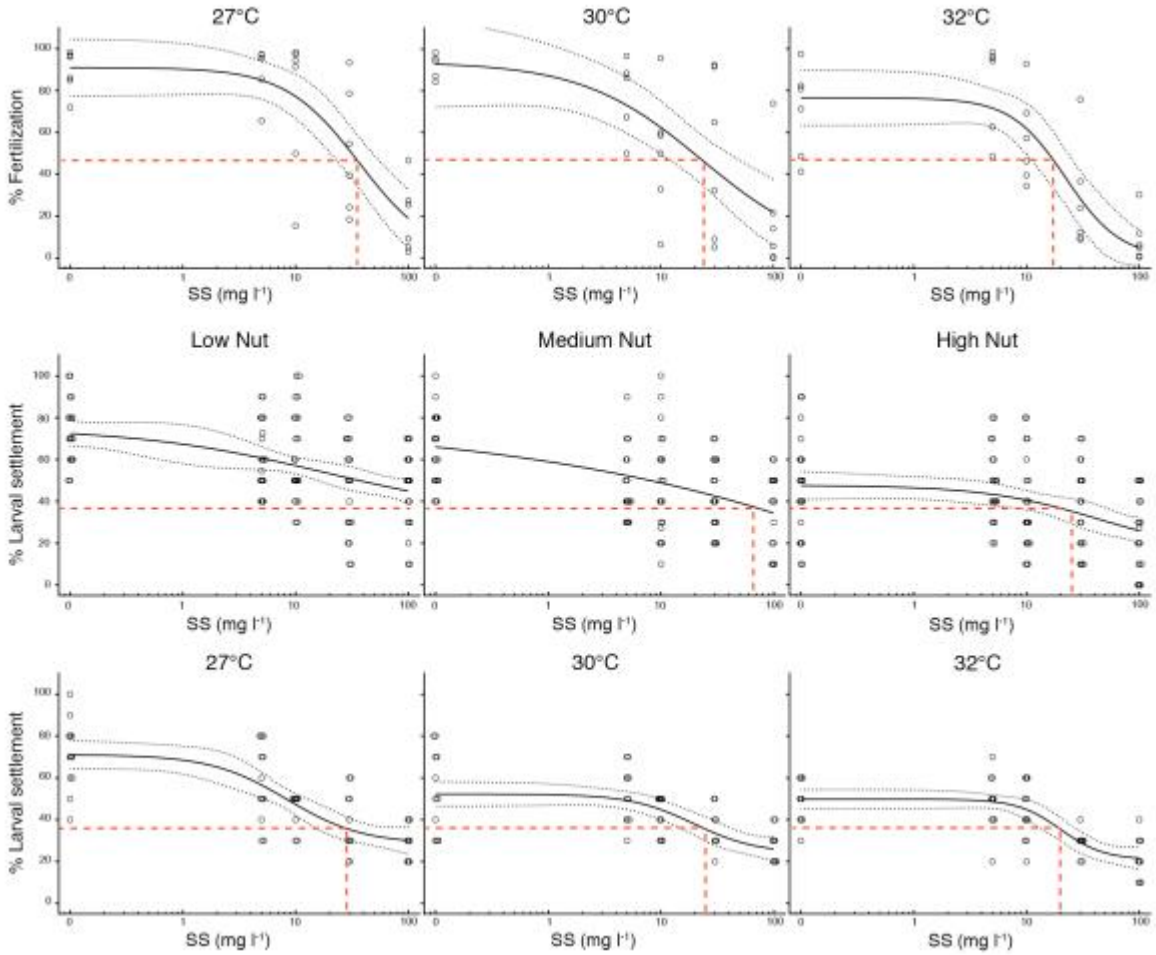

Table S9: Concentration of suspended sediments which inhibit fertilization or settlement of coral embryos or larvae respectively by 50% (IC_{50}). Absolute EC_{50} values were calculated using a four-parameter logistic model (S11 Fig). All models were interpolated to half (50%) of the Top of the control temperature (27°C) or nutrient concentration (Low). N: Normality of residuals test, R: Replicates test, H: Homogeneity of variance. P: passed, F: failed.

	**Factor level**	**SS Best-fit parameters (95% CI)**				**N**	**R**	**H**
		**IC_{50} (mg l^{-1})**	**Top (%)**	**Bottom (%)**	**Slope**			
**Fertilization (Experiment 1)**	**27°C**	36.61 (19.91–68.50)	91.05 (76.56–100.00)	0.00 (0.00)	-1.33 (-3.05–-0.69)	P	P	P
	**30°C**	25.00 (11.22–69.02)	93.83 (69.06–100.00)	0.00 (0.00)	-0.89 (-2.083–-0.23)	P	P	P
	**32°C**	17.99 (10.57–28.84)	76.57(63.03–91.98)	0.00 (0.00)	-1.79 (-∞–-0.93)	P	P	P
**Larvae settlement (Experiment 2)**	**Low**	>100	75.13 (N/A)	32.22 (N/A–100.00)	-0.51 (-2.65–2.65)	P	P	P
	**Med**	63.97 (N/A)	72.22 (N/A)	~0.00(N/A)	-0.35 (N/A)	N/A	N/A	N/A
	**High**	17.49 (3.88–54.20)	47.72 (40.42–81.57)	15.40 (N/A–35.06)	-0.86 (-∞–-0.14)	F	P	P
**Larvae settlement (Experiment 2)**	**27°C**	29.11(14.26–∞)	71.13 (63.08–N/A)	28.36 (0.00–37.59)	-1.28 (-3.93–N/A)	P	P	P
	**30°C**	24.66 (14.16–48.98)	52.28 (46.07–N/A)	23.90 (0.00–33.44)	-1.53 (-∞–N/A)	P	P	P
	**32°C**	19.82 (12.74–29.72)	49.82 (44.83–55.84)	20.90 (0.00–27.84)	-2.26 (N/A–0.71)	P	P	P

Table S10: Results of generalized linear models (GLM) of the effects of organic nutrient enrichment (Nut: low, medium, high) during gamete fertilization of *Acropora tenuis* comparing common treatments (SS: 0 mg l^{-1} and Temp: 27 °C) of this study and Humanes et al. 2016 [2]. Suspended sediments, nutrient enrichment and temperature were considered as fixed factors, significance at p<0.05 (in **bold**). Df: degrees of freedom.

Factor	Df	Deviance	F-value	p-value
Experiment	1	308.1	1.90	0.117
Nut x Experiment	2	290.8	0.52	0.598

Table S11: Gamete fertilization success, larval survival and larval settlement (mean ± s.d.) per treatment level for each experiment performed (see Fig 5 for experiment details).

	Suspended sediments (mg l^{-1})	Nutrient enrichment			Temperature		
**Experiment**		**Low**	**Medium**	**High**	**27°C**	**30°C**	**32°C**
Fertilization (Experiment 1)	0	90±5	89±4	78±16	89±10	92±6	70±21
	5	82±12	79±8	81±10	89±12	81±19	83±21
	10	78±13	72±16	70±13	75±34	51±30	57±22
	30	56±16	70±16	62±10	51±20	49±39	28±26
	100	35±11	40±20	30±14	20±17	19±28	9±11
Larvae survivorship (Experiment 2)	0	93±9	97±5	95±7	93±10	83±21	87±12
	5	98±4	94±8	97±6	83±21	85±14	89±14
	10	96±5	95±7	94±9	76±16	91±9	88±11
	30	94±8	94±8	95±7	93±10	74±13	86±13
	100	97±5	96±6	96±6	83±12	81±14	81±23
Larvae settlement (Experiment 2)	0	73±14	66±17	47±22	71±17	52±20	50±10
	5	59±16	50±16	46±17	58±19	51±13	48±13
	10	60±21	52±26	37±22	46±8	43±9	45±14
	30	49±21	42±16	35±19	37±15	34±9	29±5
	100	45±18	34±17	26±19	29±8	26±8	22±10

Table S11 (continued):

Experiment	Suspended sediments (mg l^{-1})	Nutrient enrichment			Temperature		
		Low	Medium	High	27°C	30°C	32°C
Larvae survivorship (Experiment 3)	0	80±18	67±22	84±10	84±15	93±21	86±23
	5	53±28	45±17	44±27	81±23	87±19	84±17
	10	37±25	58±30	72±23	74±32	86±19	81±31
	30	59±35	61±28	70±20	88±18	87±18	68±32
	100	85±31	89±7	66±32	88±16	83±31	88±21
Larvae settlement (Experiment 3)	0	30±23	35±21	36±25	66±22	53±29	60±20
	5	7±11	8±14	20±15	58±27	61±22	46±26
	10	27±32	15±16	27±13	54±33	62±21	41±28
	30	20±23	15±13	15±11	59±24	58±25	44±19
	100	6±7	32±19	26±16	60±21	60±31	46±26
Larvae settlement (Experiment 4)	0	46±14	48±6	26±16	62±19	72±13	51±23
	5	43±27	35±18	50±17	68±15	75±17	62±17
	10	38±21	43±24	45±7	71±19	74±16	51±12
	30	34±9	44±5	56±4	87±6	76±7	63±21
	100	39±10	44±4	57±27	85±7	71±8	60±27

S12: Experimental tank set up used for maintaining sediments in suspension used in Experiments 2 and 4

Each experimental system consisted of two polyethylene containers: 1) a sump tank (4 l), which contained an air-stone to keep the sediments in suspension and a pump to transfer the modified seawater up into the experimental tank at 20 ml s^{-1}; and 2) an experimental tank (5 l), which was at a height greater than that of the sump tank, and had an outflow pipe that operated as a siphon (Supplementary Material Fig. S13). The outflow pipe was covered with 160 µm plankton mesh and had an air-stone positioned beneath it to prevent loss of embryos.

Figure S13: Experimental tank set-up used for maintaining sediments in suspensions and expose *A. tenuis* 8-h old embryos for 28 h (Experiment 2), and 5-d-old larvae for 24 h (Experiment 4) to suspended sediments (0, 5, 10, 30 and 100 mg l^{-1}) under different nutrient enrichment (low, medium, high) or contrasting temperatures (27, 30, and 32°C). The grids were used only during Experiment 4 to suspend settlement substrata (2 cm aragonite plugs covered with crustose coralline algae) in the larval tanks. Dashed lines indicate water flow.

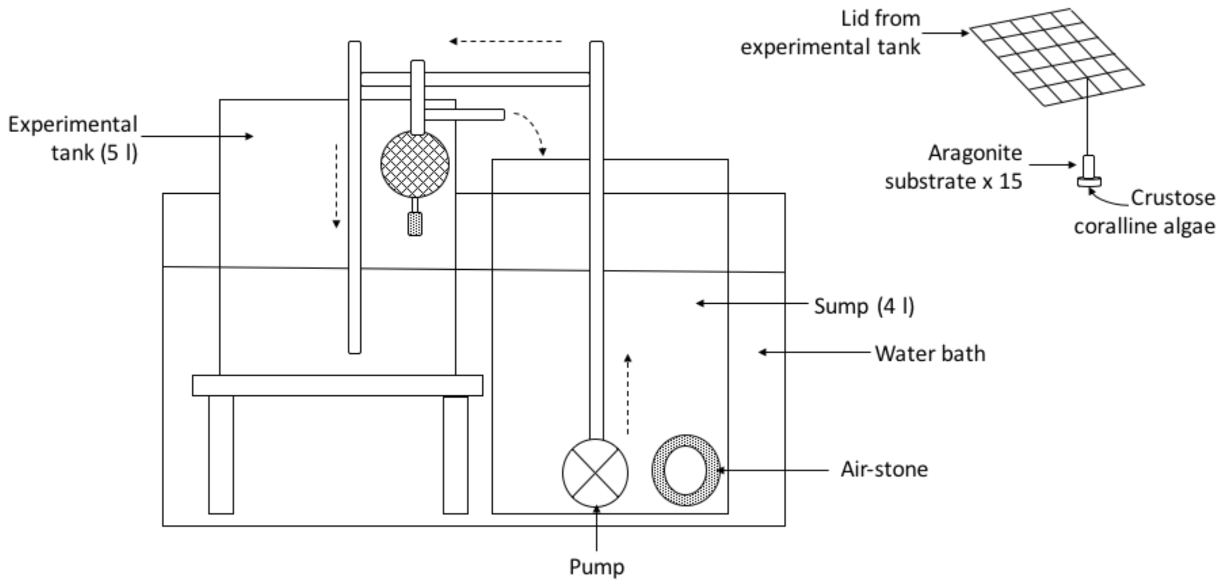

References:

1 Schaffelke, B., Carleton, J., Skuza, M., Zagorskis, I. & Furnas, M. J. Water quality in the inshore Great Barrier Reef lagoon: Implications for long-term monitoring and management. *Marine Pollution Bulletin* **65**, 249-260, doi:10.1016/j.marpolbul.2011.10.031 (2012).

2 Humanes, A., Noonan, S., Willis, B. L., Fabricius, K. E. & Negri, A. P. Cumulative effects of nutrient enrichment and elevated temperature compromise the early life history stages of the coral *Acropora tenuis*. *PLoS ONE* **11**, e0161616 (2016).
